# Self-Compassion and Caregiving Outcomes: Insights from a New Theoretical Model

**DOI:** 10.1093/geroni/igaf122.3481

**Published:** 2025-12-31

**Authors:** Claire Grant, Katherine Judge

**Affiliations:** VA, Waltham, Massachusetts, United States; Cleveland State University, Cleveland, Ohio, United States

## Abstract

An expansion of theoretical modeling of the stress process for caregivers of people living with dementia (PLWD) is essential for the advancement of theoretical and applied literatures. The proposed presentation will outline results from a recent study testing an adapted model of self-compassion in caregivers of PLWD (Cha et al., 2023). The model tests parallel lines of mediation by which self-compassion, a way of relating to oneself, influences outcomes including depression, anxiety, and positive aspects of caregiving through mediators including the general resource, emotion regulation, and coping strategies. Caregivers of PLWDs were recruited through CloudResearch and social media posts (N = 226). Participants were majority female (50%), White (70%) and were middle aged (M = 40.5, SD = 12). Findings demonstrated support for self-compassion impacting depression though emotion focused and dysfunctional coping (b=-.05,95%CI [-.0877,-.0139]); b=-.15, 95%CI [-.2150,-.0874]). The general resource (b=-.09,95%CI[-.1777,-.0018]) and dysfunctional coping (b=-.11,95%CI[-.1964,.0583) mediated the relationships between self-compassion and anxiety. Finally, self-compassion indirectly impacted positive aspects of caregiving through problem focused coping (b=.13,95%CI[.0595,.2264]). and the emotion regulation technique of cognitive reappraisal (b=.15, 95%CI[.0143,.2699]). Overall, the results highlight the role of coping as a mechanism of self-compassion to impact a range of caregiver outcomes as well as the important similarities and differences in the significant mediators and relationships. The findings may be extended in future research to further refine theoretical understanding of the caregiving process and may have implications in intervention development. Current applications of these findings as well as future implications will be discussed.

